# The Automatic Methods Group *Newsletter*

**DOI:** 10.1155/S1463924698000182

**Published:** 1998


Journal of Automatic Chemistry, Vol. 20, No. 4 (July–August 1998) pp. 121–128

AUTOMATIC METHODS GROUP

Analytical Division
ROYAL SOCIETY OF CHEMISTRY

### The Automatic Methods Group *Newsletter*

The AMG Newsletter is published as part of and in collaboration with the *Journal of Automatic Chemistry*. Further information on the *Journal of Automatic Chemistry* can be obtained from Taylor & Francis Ltd, Rankine Road, Basingstoke, Hampshire RG24 8PR or e-mail:info@tandf.co.uk; worldwide web home page: http:||tandf.co.uk.

### Conference Report

*Ambient air monitoring and the EU framework directive*

A two-day meeting from 16 to 17 December 1997, at the Scientific Societies Lecture Theatre, London was held under the sponsorship of the Automatic Methods Group and co-operation of the Health & Safety Executive. The meeting was sponsored by Perkin Elmer Ltd.

### Abstracts of papers presented

### EU ambient air directive and its impact before EU members

*Emile De Saeger*, European Commission—Joint Research Centre/Ispra—Environment Institute, European Reference Laboratory of Air Pollution

The European policy on air quality was initiated in 1980. The experience gained with the implementation of earlier directives issued in this period gave rise to various problems and inadequacies. The main problems arising in the implementation of these directives were caused by differences and inconsistencies in the reporting between various member states. This included the designation of zones of risk and plans for improvement of air quality. Moreover, there appeared to be a certain incomparability of monitoring strategies and a large variety in the quality of the analytical measurements over the area of the European Union. Finally, it became clear that a considerable length of time was required before compliance with the directives was achieved.

Therefore, the Commission adopted, in 1996, a new Framework Directive which established a more integrated and coherent approach to air quality management at the level of the European Union. The objectives of the directive are the following:

- to define and establish objectives for the ambient air quality in Europe;
- to assess the ambient air quality in the Member States on one common basis;
- to obtain a maximum of information on ambient air quality and to make it available to the public;
- to maintain and to improve the ambient air quality in all Member States.

The Directive establishes a European-wide framework for the assessment and management of the ambient outdoor air quality. The main issues addressed are:

- the list of substances which should be covered;
- the principle to establish limit and alert values for designated pollutants;
- the ways to assess air quality in a uniform and coherent way;
- public information about air quality.

To this end, a two-step approach is being applied: a Framework Directive, fixing the principles of the new strategy, followed by a series of specific ‘Daughter Directives’ on a pollutant-by-pollutant basis. The elements to be established for each pollutant are the criteria and techniques for:

- the location of the sampling points;
- the minimum number of sampling points;
- the reference measurement and sampling techniques;
- spatial resolution for modelling and objective assessment methods;
- reference modelling techniques.

0142-0453/98 $12·00 © 1998 Taylor & Francis Ltd

The Commission has recently submitted the Daughter Directive proposal for $SO_2$, $NO_2$, PM10 and Pb to the Council of Ministers. The development of other Daughter directives for $O_3$, benzene, CO, PAH, Cd, Ni, As and Hg has been scheduled by 2000.

### Monitoring airborne particulates: PM10, PM2.5?

*A. van der Meulen*, National Institute of Public Health and the Environment (RIVM), PO Box 1—3720 BA Bilthoven, The Netherlands

In order to harmonize monitoring methods for ambient suspended particulate matter (SPM), in the EU daughter directive on PM10 (SPM below 10 µm) the following approach, laid down in CEN standard EN12341, has been adopted:

- the recommendation of a PM10 reference method;
- the obligation to use the reference method under routine conditions for the survey of ambient air quality, or methods which are equivalent to the reference method;
- the obligation to take the necessary steps to show that equivalence methods are tested by an appropriate reference equivalence procedure.

Separate PM10 reference instruments are suggested for the Low-, High- and SuperHigh Volume flow regime respectively. For the Low- and High-Volume flow regimes, *LVS-PM10* and *HVS-PM10* samplers are recommended, following the requirements in CEN standard EN12341 concerning specific inlet design, flow rate performance, and gravimetric (handling, conditioning and weighing) procedures. As a rule this implies the application of commercially available non-automatic *LVS-PM10* or *HVS-PM10* samplers. The WRAC serves as reference instrument for the SuperHigh Volume flow regime; however, the practicability of the WRAC system is a major drawback. The performance of the reference samplers turned out to be significantly better than the appropriate requirements from CEN standard EN12341. In particular, high flow rates and/or electronic flow control were found to result in excellent comparability.

Reference equivalence of candidate PM10 samplers can be obtained for prevailing characteristic situations in the European countries. The field test procedure given in CEN EN12341 only secures the reference equivalence to hold over the range of these characteristic situations ('interpolation') and not outside this range (i.e. 'extrapolation' is not permitted). The testing efforts are of limited, reasonable proportions, provided the technical competence of the testing laboratories is in accordance with the general criteria specified in EN45001.

In the practice of air monitoring the TEOM- (Tapered Element Oscillating Microbalance) and Beta-monitoring systems are widely applied. However, following the test procedures from CEN standard EN12341 these automatic PM10 samplers seem to be of limited suitability. To stabilize the TEOM oscillating element, it is commonly heated up to approximately 50°C, as is the inlet tubing of the Beta-system to eliminate humidity. As a consequence, these sampling systems are measuring lower concentrations with respect to the reference sampler, presumably due to losses of semi-volatile compounds (especially ammonium nitrate); e.g. in the city of Madrid both the TEOM- and Beta-monitoring system are equivalent to the reference sampler. However, in The Netherlands or Berlin these systems showed a consistent underestimate of *c.* 30%. Moreover, as a rule, automatic monitoring systems are equipped with (very) long inlet tubings, possibly giving rise to particle losses, i.e. lower concentrations compared to the reference sampler.

When it comes to PM2.5 sampling, it is to be expected that the loss of semi-volatile compounds is playing an increasing role, in view of the increased relative contribution of ammonium nitrate aerosol.

### Air quality—diffusive monitoring to meet requirements of the directive

*Richard H. Brown*, Health and Safety Laboratory, Health and Safety Executive, Broad Lane, Sheffield

Diffusive sampling is particularly relevant to Articles 5 and 6 of the EC Directive on Ambient Air Quality Assessment and Management (96/62/EC), which will, via Daughter Directives, extend the list of atmospheric pollutants to be regulated against new pollution indicators. The framework and Daughter Directives will also allow for the use of non-continuous measurement techniques for the monitoring of air quality, provided they meet the relevant data quality objectives. Of these techniques, diffusive sampling is ideally suited, because of its low cost and ease of deployment at multiple locations, to serve the indicative and possibly also the mandatory measurement requirements in a number of specific areas of the Directive: tool for the siting of network stations (Art. 4.3); preliminary assessment of ambient air quality (Art. 5); air quality monitoring in areas not exceeding limit values (Art. 6.3); and classification of zones (Art. 8 and 9).

Not all pollutants covered by existing directives (sulphur dioxide, nitrogen dioxide, fine particulate matter (PM10), suspended particulate matter, lead and ozone) or added to in the Framework Directive (benzene, polyaromatic hydrocarbons, carbon monoxide, cadmium, arsenic, nickel and mercury) are amenable to diffusive sampling. In principle, any that are volatile are suitable, provided sufficient sensitivity can be achieved.

As a result of this potential of diffusive sampling to measure volatile pollutants in support of the Directives, a working group of CEN TC 264 has been set up to develop performance criteria for such samplers. The rationale behind WG11's approach has been borrowed from CEN/TC137 (the workplace air quality equivalent to TC 264), which has established general performance requirements for measurement methods (EN 482) together with specific requirements for diffusive samplers (EN 838). The actual establishment of measurement procedures (meeting the TC 137 requirements) is the province of ISO. Parts 1 and 2 of the WG 11 standard closely parallel EN 482 and EN 838, and are simply adapted for ambient air monitoring. Part 3 of the standard is a guidance document on the practical use of diffusive samplers. On the basis of parts 1 and 2 of the WG 11 standard, it will be possible to evaluate diffusive samplers for their suitability as indicative and/or mandatory measurement procedures in support of the Directives, and allow the Commission to establish reference methods, where appropriate. The methods themselves can then be elaborated by the working groups of TC 264 (e.g. 12 and 13) or by ISO.

### Reference methods for sulphur dioxide, nitrogen oxides, carbon monoxide and ozone

*Ente Sneek*, Convenor CEN TC 264 WG 12, KEMA, Plant & Systems Performance Monitoring, The Netherlands

In 1996, within CEN TC 264 'Air Quality', dealing with standardization of measurement methods for a.o. air pollution compounds in ambient air, a working group was established to prepare reference methods for the determination of sulphur dioxide, nitrogen oxides, carbon monoxide and ozone. The first idea was that the work could be done rather easily, because of the existence of ISO standards in the field of ambient air measurements and the available experimental data about comparative measurements and analyser performance.

During the first year the work was slow because the requirements for the reference methods, set up by the European Commission, appeared to be still in development. This was mainly caused by the fact that the Daughter Directives, in which the reference methods will be specified, are not yet fully accepted in the European Union. It now appears that the reference methods, as specified by the Commission shall have to deal with the following items:

- the range of application (concentration levels for which the method applies);
- a description of the method concerning sampling, calibration aspects and analysis;
- an overview of the relevant performance characteristics; and
- the values of performance characteristics of the method.

Discussion regarding whether the following subjects also have to be incorporated in the reference method is still on-going:

- the test procedures to establish the performance characteristics and the overall uncertainty;
- recommendations for use in field operation and relating to QA/QC aspects.

A short overview was given on the discussions in the working group, which led to a preliminary choice of the measurement principles for the four compounds.

### Reference monitoring methods of benzene

*Henrik Skov*, National Environmental Research Institute, Frederiksborgvej 399, DK-4000 Roskilde, Denmark

Benzene is a highly carcinogenic compound. A benzene concentration of 1.7 $\mu g/m^3$ is estimated to be associated with an excess lifetime risk for leukaemia of 1/100 000. Therefore, benzene is included in the new ambient air directive where a limit value for benzene of 10 $\mu g/m^3$ in ambient air is proposed. In order to execute this directive, it is important that measurements of benzene throughout Europe are comparable. For this reason a working group, WG 13 under CEN/TC 264, is mandated by the commission. The mandate is to prepare a method for benzene measurements in ambient air, including test procedures for alternative methods.

The standard method is selected by the EU-Commission (DG XI). It is likely that pumped sampling on active cartridges followed by extraction and GC analysis will be chosen. In practice this method includes several different techniques: one group is manual where ambient air is pumped through cartridges placed in the field, which afterwards are brought to the laboratory, extracted and analysed by GC, another group measures benzene *in situ* by automatically sampling, thermal desorption followed by GC analysis.

The verification of the different methods in WG 13 is divided into three stages:

(1) Review of available methods for monitoring benzene.
(2) Identification of standard methods in accordance with DG XI decision and development of a test protocol, including guidelines for verification of equivalent methods.
(3) Establishment of the performance of the standard methods in accordance with the DG XI decision.

Currently WG 13 works on (1) and (2).

The techniques applied by the different countries were summarized. For each method the detection limits, range of application, advantages and disadvantages were described. A test protocol was elaborated which included a test of detection limit and concentration range of relevance for Europe (0–500 $\mu g/m^3$), as well as precision and accuracy. The accuracy was determined under controlled laboratory conditions with a traceable standard (e.g. NIST standard) and the effect of possible interfering species was also investigated. Finally, the robustness of the method was carried out in the field. Based on the above described work a standard for benzene in ambient air was elaborated.

### Lead, cadmium and mercury—measurements in ambient air

*Klaus Berger*, ERGO Hamburg, Germany

The European Council Directive 96/62/EC on Ambient Air Quality Assessment and Management (Air Quality Framework Directive) lists in Annex I air pollutants to be taken into consideration in the assessment and management of ambient air quality. In this list lead, cadmium, nickel, arsenic and mercury are the toxic metals for which limit values shall be established.

According to the Air Quality Framework Directive the measurement of particulate lead, cadmium, arsenic and nickel in ambient air is demanded to certify the compliance with the limit values. As there are, so far, no national standards available for the determination of the four heavy metals in suspended particulate matter below 10 $\mu m$ (PM 10 fraction), there is a great demand for an European standard for use as a reference method for this purpose. At the sixth plenary meeting of CEN/TC 264 'Air Quality', held in Rotterdam, it was agreed to establish WG 14.

CEN/TC264 WG 14 is developing a standard that describes a measuring method for the determination of lead, cadmium, arsenic and nickel in aerosols in ambient air, which can be used as a reference method in the Air Quality Framework Directive and the Daughter Directive, e.g. the Position Paper on lead. According to the Position Paper on lead the sampling was carried out with equipment that gave equivalent results as sampling for PM10. The sampling devices were tested in accordance with EN 12341 (procedure for testing sampling devices for PM 10).

The EN Standard will set minimum requirements for certain key parameters, e.g. the sample filter material. Atomic absorption spectrometry (AAS) and inductively coupled plasma (ICP) were described as the basic techniques for analysis.

In order to choose the correct filter material blank values, recovery rates of the four elements and the limits of the analytical method, as well as the pre-treatment method (digestion) were determined in the laboratory with certified reference materials (CRMs). The comparative measurements were carried out to check the equivalence of the results obtained with the different measurement methods used for the validation tests. These measurements were also required to detect problems with the sampling technique and also the sampling material when used for field measurements. For the information on the repeatability and reproducibility of the reference method interlaboratory field tests are necessary.

Until November 1997 no mandate was given by the EU to develop the prescribed standard. Mercury will be a subject for another standard, but until now here is no working group established dealing with the measurement of mercury in ambient air.

### The use of thermal desorption in EPA projects and methods

*R. K. M. Jayanty, M. R. Peterson, and G. B. Howe Research,* Triangle Institute, Research Triangle Park, North Carolina, USA

Accurate measurements of atmospheric concentrations of volatile organic compounds (VOCs) are necessary in the application of photochemical models that are used in developing control strategies needed to achieve ambient air quality standards for ozone and photochemical oxidants. Two general approaches to ambient measurements are currently in use—continuous (on-site) sampling and analysis and on-site sampling with laboratory analysis. Measurements obtained with continuous on-site analysers have shown limited reproducibility due to a variety of problems. On-site sampling with laboratory analysis methods provides organic measurements representative of samples collected over a specific time period.

Several approaches have been utilized for sampling and analysis of VOCs in air. Sample collection methods for target compounds include adsorption on solid sorbents (e.g. Tenax®, XAD-2 and charcoal), condensation in a cryogenic trap and whole-air sampling using some type of container (bags or stainless steel containers). The principal limitation of whole-air sampling is that it does not provide sample preconcentration, which affects both sampling volume and detection limits. During the last 15 years, the collection of VOCs on solid sorbents followed by thermal desorption and gas chromatographic analysis with a selective detector has become one of the most widely used methods for ambient air monitoring.

Staff of the Research Triangle Institute (RTI), under contracts to the U.S. Environmental Protection Agency (USEPA) and commercial clients, have evaluated several test methods for measuring concentrations of VOCs (either as total VOC or speciated compounds) present in ambient air. Methods evaluated for monitoring VOCs in ambient air include EPA Compendium Methods TO-12, TO-14, and TO-17. EPA Methods TO-12 and TO-14 involve the collection of whole air samples in passivated canisters followed by gas chromatographic (GC) analysis for the determination of total nonmethane organic compounds (NMOC) or individual VOCs. EPA Method TO-17 involves pumped sampling of ambient air through sorbent tubes with analysis by thermal desorption and capillary gas chromatography.

Other thermal desorption methods evaluated at RTI include methods for measuring total VOC content and for measuring individual hazardous air pollutants (HAPs) emitted during the curing of surface coatings. Automated thermal desorption with flame ionization detection (ATD-FID) and an ATD with oxidation, separation of oxidized species, and thermal conductivity detection (TCD) of $CO_2$ were evaluated as means for measuring total VOCs emitted from coatings. An ATD-GC-FID approach for measuring target HAPs emitted during curing of coatings was also evaluated. The experimental details and the results obtained with each of the above methods were described.

### Pumped vs diffusive sampling

*Neil T. Plant,* Health and Safety Laboratory, Health and Safety Executive, Broad Lane, Sheffield

Diffusive sampling is particularly relevant to Articles 5 and 6 of the EC Directive on Ambient Air Quality Assessment and Management (96/62/EC), which will, via Daughter Directives, extend the list of atmospheric pollutants to be regulated against new pollution indicators. The framework and Daughter Directives will also allow for the use of noncontinuous measurement techniques for the monitoring of air quality, provided they meet the relevant data quality objectives. Of these techniques, diffusive sampling is ideally suited, because of its low cost and ease of deployment at multiple locations, to serve the indicative and possibly also the mandatory measurement requirements in a number of specific areas of the Directive: tool for the siting of network stations (Art. 4.3); preliminary assessment of ambient air quality (Art. 5); air quality monitoring in areas not exceeding limit values (Art. 6.3); and, classification of zones (Art. 8 and 9).

A particularly attractive candidate for ambient air monitoring by diffusive sampling is benzene. This can be sampled readily by using sorption tubes, thermal desorption and gas chromatography; a method which has been fully validated for workplace air monitoring. Much longer sampling times are required for ambient air monitoring, and it is necessary as part of the validation process to establish reliable diffusive uptake rates for periods of up to four weeks. This has been done at the HSL laboratory, comparing pumped and diffusive sampling operating simultaneously. The actual concentration of benzene (and toluene and xylene) in ambient air in the vicinity of the HSL building has been established by sequential pumped sampling and the diffusive sampling rates of parallel diffusive samplers determined from these concentrations.

A second feature of a full validation of the environmental diffusive sampler is a comparison with fixed monitoring instruments employing semi-continuous VOC air analysers as used in the UK monitoring stations. One such intercomparison was done with the co-operation with the relevant local authorities around March 1997. There were some difficulties with the data capture efficiency of the VOC air analysers, partly because of the changeover of responsibilities for managing the network. However, where there was a reasonably complete data set, diffusive and pumped samplers gave qualitatively similar results. Specific conclusions from a quantitative comparison will be presented at the meeting.

### Making thermal desorption work for 1,3-butadiene: issues and developments

*Alexander P. Bianchi*, Exxon Chemical Co.

1,3-butadiene (BD) is a highly volatile, colourless, noncorrosive gas with a mild aromatic and gasoline odour, and a boiling point of −4.4°C. BD is a known carcinogen in rats and mice and a suspect human carcinogen (EU Class. Category 2). The BD molecule requires metabolic activation to DNA-reacting epoxides that can bind to NA to initiate events leading to tumour formation.

BD is a monomer widely used in the manufacture of synthetic rubber (e.g. styrene–butadiene rubber) and other resins. It causes low acute inhalation toxicity in animals and sensory irritation in humans. Occupational exposures to butadiene occur during production, storage and transport of the material. The current UK OEL is set at 10 ppm (8-hr TWA) although many other major organizations which handle BD have set operational and legal exposure limits at much lower levels (e.g. < 2 ppm), as have some regulatory authorities, to reduce the likelihood of chronic health risks. BD is also found in cigarette smoke in indoor locations and is also ubiquitous in many city, urban and related environments as a component of vehicle exhaust gases and emissions.

In the UK, the Expert Panel on Air Quality has set a health-based limit of 1 ppbv (running annual average) for BD, presenting technology challenges for air monitoring techniques. Often, sampling BD requires the use of suitable preconcentrating methods, followed by quantitative, 'interference-free' retention of the material on an inert adsorbing medium. Historically, problems have been encountered with breakthrough, inadequate adsorption, method reproducibility, poor stability, and in obtaining satisfactory limits-of-detection (i.e. sensitivity).

Thermal desorption methods offer major technical advantages to the environmental or occupational health chemist, although the technique is not without its own challenges. Nonetheless, when subjected to critical comparisons with alternative 'activated-charcoal' based methods during a recent European chemical-industry led exercise (i.e. CEFIC), pumped sampling and thermal desorption methods (and the resultant data) were found to be more accurate, precise and reliable than past methods utilizing pumped or diffusive sampling onto charcoal wafers or charcoal-packed tubes. These conclusions, published in a 1997 CEFIC report, have significant implications for toxicological assessments of dose–effect relationships surrounding BD.

The integral advantages (and disadvantages) offered by thermal desorption methods were discussed in the context of modern, relevant environmental and health-based requirements, and method applications.

### The influence of the chromatography on thermal desorption methods

*Greg Johnson*, Perkin Elmer Ltd, Beaconsfield, England

Gas chromatography is an analytical technique used for the investigation of both volatile and semi-volatile compounds. James and Martin first demonstrated the technique, for the analysis of volatile fatty acids, in their classic paper published in 1952.

Gas chromatography encompasses the use of both gas–liquid (GLC) and gas–solid (GSC) based separations. A vaporized aliquot of sample is introduced into an inert mobile phase and transferred to a chromatographic column. Components of the sample are separated here, by means of their selective interaction (partitioning in GLC or adsorption in GSC) with the contents of the column. While thermal desorption methods almost invariably depend on GSC type interactions, to both collect and then desorb the ample components, their subsequent separation may use either, or in some cases, both forms of chromatography.

Within the conference proceedings, participants heard details concerning the development of methods for automated thermal desorption, or saw the results of such development. Gas chromatographic separation and specifically, how that aspect of the method development, interacts with thermal desorption methodology, was dealt with.

Gas chromatography presents the analyst a wealth of variables to be optimized. The choice of detector and the resulting limit of detection is only one consideration. In developing an overall strategy, it may be generally preferable to choose capillary GC-MS, as the optimum means of separation and detection, but if this proves impossible, compromise solutions must be sought.

Although not intended as a definitive work on the subject, the typical logic used in developing chromatographic methods, for use with thermal desorption was demonstrated. Several examples were used to illustrate, for example, when packed or capillary columns are the best choice and when specialized techniques, such as multidimensional gas chromatography should be considered. Consideration was also given to how choices between diffusive and pumped sampling influence the chromatographic separation. Particular emphasis was given to how the adsorbent used for sample collection influences the choice of parameters, e.g. the column phase ratio.

### Automated hydrocarbon measurements to monitor UK urban air quality

*Paul Quincey*, National Physical Laboratory, Teddington, Middlesex TW11 0LW, UK

The UK Hydrocarbon Network forms part of the set of activities by which the Department of the Environment, Transport and the Regions monitors UK air quality and disseminates the information to the public. There is emphasis throughout all the networks on traceable, consistent measurements giving representative geographical coverage over periods of many years.

The hydrocarbon measurement system, at 12 sites throughout the UK, produce a gas chromatogram every hour. Thirty-minute average samples of ambient air are taken by pumping it through a trap cooled to −20°C, containing three consecutive absorbing materials. At the end of the sampling period the hydrocarbons are removed from the trap by heating, and collected at a point on the GC column cooled to −99°C. The hydrocarbons are then released into the column by flash heating.

The site instruments are all automated and connected via modem to a central unit, which interrogates them hourly to collect the raw chromatogram data and provisionally processed concentration data. These data for benzene and 1,3-butadiene are disseminated rapidly, with data from other DETR networks, to Ceefax and web pages. Provisional data for 25 species are available on the archive web pages (http://www.aeat.co.uk/netcen/aqarchive/auto.html). The principal mechanism for ensuring data accuracy is the regular (fortnightly) use of well-characterized calibration mixtures. These have been made at NPL gravimetrically, with known masses of each of 27 hydrocarbon components, and the balance gas, in specially prepared cylinders, and their stability and consistency have been carefully checked. They are injected into the sampling line at each site and therefore calibrate the entire system with the exception only of the sampling line ahead of the injection port, in which the ambient air is kept moving at a substantial flow rate in large diameter pipe to avoid significant hydrocarbon losses.

The final 'ratified' data are produced from the raw chromatograms some three months in arrears, using more sophisticated peak identification software, making full use of all relevant calibration information, and with human intervention in particular cases of problems such as coelution of peaks and wandering artefacts. The ratified data are incorporated into the archive, where it replaces the provisional data, as it becomes available.

### Thermal desorption instrumentation—recent developments

*Kaj Petersen*, Perkin Elmer Ltd, Wilton, USA

Recent developments in instrumentation hardware and control software will enable a further extension of the application range of thermal desorption analysis. In particular, an increase in the internal temperature range of the ATD 4000 automated thermal desorber makes possible the analysis of higher-boiling analytes such as polyaromatic hydrocarbons (PAH). Examples of this type of analysis performed as part of studies for European Union projects were shown. Data included chromatograms, recovery and carry-over for PAH compounds. Also, examples of the analysis of polychlorinated biphenyls (PCB) were presented. The new instrument features of the ATD 400 Thermal Desorber, upon which the extended application range is based, were presented and discussed. These included improved and extended thermal range, and thermal insulation in the areas of the heated valve and the internal tubing connecting to the cold trap, as well as improved internal temperature control. To cover internal temperatures higher than the presently allowed maximum temperature of 225°C, a polyimide-based valve rotor was being used.

A control software for the ATD 400 automated thermal desorber, which operates on PC under Windows 95™ or Windows NT™, was presented. A highly graphical user interface allows easy set-up of sequences and methods and enabled the analyst to control the thermal desorber entirely from a PC without the need for a separate external keypad. The ATD 400 Control Software offers the analyst simplified operation, added flexibility and improved control for tube desorption analysis, as well as for automated on-line air monitoring. Up to 99 methods can be linked, one or more of which can be assigned to any of the available 50 tube positions. This newly introduced flexibility enables redesorption of samples at varying temperatures for more efficient method development. Also, if necessary, tubes can be automatically conditioned after analysis in one automated sequence. Sequences can be paused and modified allowing free movement of the carousel for addition of tubes. Extensive documentation of analysis parameters and instrument performance offers the benefit of improved tube tracking for GLP purposes, allowing the analyst to take full advantage of unique ATD 400 sample protection features such as the pre-analysis tube leak test. The ATD 400 Control Software has been prepared for external communication allowing external control of the instrument from an automated sequence including transfer of tube numbers to the data handling program.

Newly developed internal control-firmware for the ATD 400 was presented, which adds flexibility in tube purging and tube method assignment. To allow improved, automated simulation of materials outgassing, extended desorb time and trap hold time have been implemented. A programmable purge time for the desorption tube carrier gas purge allows further flexibility in pre-analysis purge of oxygen and other interfering compounds. Start-up routines and autoresume after power failures have been improved to ensure maximum productivity under all conditions.

### Monitoring indoor air quality

*Derrick Crump*, Building Research Establishment, Watford, Herts WD2 7JR, UK

People in the UK typically spend 90% of their time in indoor environments and the majority of this time is spent in the home. Non-occupational exposure to air pollutants is dominated by that inhaled in the indoor environment even though the main source of the pollutant may be outdoors. Volatile organic compounds (VOCs) are one type of air pollutant for which there are both indoor and outdoor sources. The potential health effects of exposure to VOCs include an increased lifetime risk of cancer, irritative effects and sensory and other effects on the nervous system.

There is a growing interest in the measurement of VOCs in air for a number of objectives including determination of the distribution of VOC concentrations and personal exposures, measurement in response to health and comfort complaints in buildings, testing of compliance with indoor air quality guidelines, identification and testing of VOC sources, and evaluation of remedial measures.

Examples of applications of the above include the BRE study of indoor air quality of homes in the Bristol area (1), monitoring of personal exposure (2), investigations of air quality problems (3) and a study of sources of VOCs in four newly built houses (4). These studies and others have used collection of VOCs in air with solid sorbents and analysis by thermal desorption and gas chromatography. To date there has been little guidance in the form of standards or other recommended procedures for planning and undertaking such studies. This is changing rapidly with recommended procedures published within the European Collaborative Action (ECA) on indoor air quality and standards under development in European (CEN) and International (ISO) standardization bodies. This paper reports progress with these standards and guidelines including those concerned with pumped sampling of VOCs in indoor air and the measurement of emissions from building products.

### Pollutants in ambient air, trace level monitoring for EU directives: comparison of available methods

*Pascual Pérez Ballesta*, European Commission—Joint Research Centre/Ispra—Environment Institute, European Reference Laboratory of Air Pollution

Over the last few years, pollution by organic compounds has been often discussed in the framework of the European ambient air quality policy. This concern has been reflected, for instance, in the EC directive 72/92 on air pollution by ozone where the measurements of ozone precursors is recommended.

The possible introduction of a VOC Directive has encouraged the Commission to initiate work on:

- the evaluation of the state-of-the-art of VOC measurement techniques in Europe by the organization of laboratory and field intercomparison exercises;
- the establishment of reference methods for sampling, measurement and calibration; and
- the definition of guidelines and quality assurance programmes for the measurements in air quality networks.

As such, the European Reference Laboratory has organized several EU intercomparisons of VOC measurements ('91/'92 and '94/'95) and a field intercomparison ('95/'96) where different monitoring techniques were tested simultaneously in a network station. These exercises have brought to light some critical aspects regarding the performance of the techniques involved: stability of the samples in the canisters and/or the cylinders; use of response factors; limits of quantification and uncertainties related to the level of concentration; grade of agreement between different techniques; and problems related to particular instrumentation.

In conclusion, the following points can be noted:

- Stability problems in cylinders were especially important for ethyne and isoprene, and to a lesser extent for the heaviest compounds C6–C9.
- Peaks merging and integration problems can be the cause of some inaccuracies in the analysis of C2 to C9 hydrocarbons.
- The use of calibration factors determined in the same concentration range as the measurements is recommended.
- The uncertainty of the measurement increases with a decrease in concentration; this being especially important for concentrations lower than 1 ppb.
- In general, acceptable correlations were found between the different techniques involved in the field exercise: on-line GC analysers, pumped tubes, diffusive samplers, canisters and TNMHC.
- The selection of a measurement technique should be linked to its monitoring objectives by taking into account the temporal and spatial resolution of the technique and the goal of such measurements in the context of global pollution and health effects on the population.

### Programme of future meetings

| | | |
|---|---|---|
| 21 October 1998 | Laboratory software and data validation | London |
| 25–26 November 1998 | Genetic analysis—its advantages and potential | Teddington |
| 14–15 December 1998 | Thermal desorption into the millennium | London |

Further information about any of these meetings can be obtained from Derrick Porter, Hon. Secretary of the Automatic Methods Group, Willowfold, Fir Tree Lane, West Chiltington, West Sussex RH20 2RA, Tel: 01798 81 2383, e-mail: amg@argonet.co.uk.

### The Automatic Methods Group's web site

Up-to-date information about the Group's activities can be obtained from the Group's web pages at:

http://www.rsc.org/lap/rsccom/db/amgmtgs.htm

Alternatively, you can access The Royal Society of Chemistry's web site at:

http://www.rsc.org and follow the links through.

Another site well worth visiting is the Chemical Societies' Network. Its home page will be found at:

http://www.chemsoc.org

### A new name to reflect evolutionary changes over the past 30 years—rationale for the change in name

The group has been called the Automatic Methods Group since it was formed in 1965.

The intervening years have seen substantial progress in analytical instrumentation. At the time of the Group's formation, analyser sequencing was usually controlled by cam-operated switches driven by synchronous motors, results were displayed on chart recorders, and data handling and data evaluation were usually carried out manually. If computing was used at all, it would have been carried out off-line on a mainframe computer. The formation of the Group in 1965 coincided with the production by DEC of their PDP 8—the first mass-produced general purpose mini-computer. The micro-computer had not yet been invented. Six years later Intel produced the first microprocessor (a 4-bit device called the 4004), to be followed two years later by the 8008, the forerunner of a range of microprocessors that eventually led to the development of the microcomputer in the 1980s.

In the early days, in 1969 and 1970, the Group held meetings at which members demonstrated mechanized or automated equipment that they had constructed. Some of these ideas were taken up and developed into commercial products by instrument companies. Such meetings would not be possible today. The emphasis is less on the mechanics of the process and more on sample tracking, information pathways, and the use that can be made of derived information for management purposes.

Present day instrumentation is highly sophisticated by the standards of 1965 and contains levels of 'intelligence' that were not even contemplated in 1965. Many incorporate multiple microcomputers that are far more powerful than mainframe computers of that time. Integral systems to self-calibrate and self-validate are becoming more common. In addition, many laboratories now have laboratory information management systems (LIMS) which typically provide a service to both the analyst and the laboratory manager. Such a LIMS can communicate with instruments to download method parameters and access raw data. Such capabilities are integrated with sample tracking, and the evaluation of QA and GLP information following QMS (quality management systems) protocols. A LIMS can provide basic data-editing, data-display and data-archiving facilities. It can document, summarize and assist in the control of laboratory resources. A LIMS systems can bridge the gap between laboratory activities and the corporate financial and administrative mainframes.

These changes have been reflected in the content of meetings organized by the Group. We still organize meetings that examine particular automation techniques in depth, but increasingly our meetings are concerned with all aspects of the organization and management of laboratories. Our meetings recognize that modern laboratories use a wide range of automated equipment coupled with advanced techniques of data handling, quality management and information management to the benefit of laboratory management in general.

Our proposal that the Group should be renamed, and the new name should be 'The Automation and Analytical Management Group', has received formal approval by the Councils of the Analytical Division and of the Royal Society of Chemistry. The new name will be brought into use as soon as the necessary formalities have been completed in collaboration with the Charity Commissioners.

Derrick G. Porter, Hon. Secretary, Automatic Methods Group, Royal Society of Chemistry, email amg@argonet.co.uk, tel. +44 179881 2383.

